# Evaluation of public subsidy for medical travel: does it protect against household impoverishment?

**DOI:** 10.1186/s12939-018-0726-z

**Published:** 2018-03-06

**Authors:** Mariyam Suzana, Helen Walls, Richard Smith, Johanna Hanefeld

**Affiliations:** 1 Faculty of Health Sciences, The Maldives National University, Handhuvaree Higun, Malé, Maldives; 2 Department of Global Health and Development, London School of Hygiene & Tropical Medicine, Keppel street, London, England WC1E 7HT

## Abstract

**Background:**

In resource-constrained health systems medical travel is a common alternative to seeking unavailable health services. This paper was motivated by the need to understand better the impact of such travel on households and health systems.

**Methods:**

We used primary data from 344 subsidized and 471 non-subsidized inbound medical travellers during June to December 2013 drawn from the North, Centre and South regions of the Maldives where three international airports are located. Using a researcher-administered questionnaire to acquire data, we calculated annual out-of-pocket (OOP) spending on health, food and non-food items among households where at least one member had travelled to another country for medical care within the last year and estimated the poverty head count using household income as a living standard measure.

**Results:**

Most of the socio demographic indicators, and costs of treatment abroad among Maldivian medical travellers were similar across different household income levels with no statistical difference between subsidized and non-subsidized travellers (*p* value: 0.499). The government subsidy across income quintiles was also similar indicating that the Maldivian health financing structure supports equality rather than being equity-sensitive. There was no statistical difference in OOP expenditure on medical care abroad and annual OOP expenditure on healthcare was similar across income quintiles. Diseases of the circulatory system, eye and musculoskeletal system had the most impoverishing effect – diseases for which half of the patients, or less, did not receive the public subsidy. Annually, 6 and 14% of the medical travellers in the Maldives fell into poverty ($2 per day) before and after making OOP payments to health care.

**Conclusion:**

Evidence of a strong association between predominant public financing of medical travel and equality was found. With universal eligibility to the government subsidy for medical travel, utilization of treatment abroad, medical expenditures abroad and OOP expenditures on health among Maldivian medical travellers were similar between the poor and the rich. However, we conclude mixed evidence on the linkages between public financing of medical travel and impoverishment which needs to be further explored with comparison of impoverishment levels between households with and without medical travel.

## Background

Eradicating poverty and inequality is one of the fundamentals of the Sustainable Development Goals (SDGs). The World Health Organization (WHO) estimates that a hundred million people are pushed into poverty as a result of payments to health care and a billion people suffer each year because they cannot access the health care they need [1]. Medical travel can help people acquire health services inaccessible in the domestic health system because of long waiting times, legal restrictions, cost of care or simply because of unavailability of the service. Although much of the demand for medical travel comes from the rich, ease of travel, targeted insurance packages and comparatively cheap prices offered by destination countries have lured the less wealthy into this industry. For example, one study of external opportunities and threats, and internal organizational strengths and weaknesses of Asian medical travel destinations, found that low-cost was a common selling point of medical travel destinations, while India especially positioned itself as providing a more holistic approach to medical services [2].

Out-of-pocket (OOP) payments to health care have shown to be high among vulnerable population groups such as migrants [3], specific disease groups [4] and in conflict areas [5]. OOP spending is widely believed to be a poor way to finance health care as it falls disproportionately on the most vulnerable, when they often need the service the most [6]. On the other hand, government financing of health care and public infrastructure in the health system has shown to be a protective factor in reducing impoverishment [7]. Medical travelers fall out of both of these protection measures as most medical tourism services are provided by private health facilities and financial protection is limited for services sought out of the local health system or the public health system. Even where overseas treatment is subsidized by governments, much of the indirect costs are borne by the households. Empirical evidence on the payments to healthcare and its impact on this group of people who seek health care across borders are lacking.

In this paper, we explored the situation in the Maldives where universal health care (UHC), through a subsidy for treatment abroad for selected diseases, has been in place since 2012. Under the Nation Social Health Insurance Act (2011) the National Social Protection Agency (NSPA) is mandated to provide free healthcare for all Maldivians. The public subsidy is provided for direct medical costs for a pre-approved set of services available in the country. Health services sought outside the country under the scheme have to be sanctioned by a public sector physician. According to health financing by function, the National Health Accounts of the Maldives 2011 showed that the largest proportion of health finance was spent on foreign providers (23.7%). Being an import-oriented economy, the Maldives imports health professionals, medical equipment, medical consumables and medical education, and travel for medical treatment is common. The Maldivians Travelling Abroad Survey 2013 by the Central Bank of the Maldives indicated that half of all Maldivians travelled abroad (MTA) for different purposes, spending a total of US$191 million in outbound travel [8]. More than 50% of this expenditure was for medical treatment abroad according to the 2016 MTA Survey. We analyzed consumption patterns of medical travelers and compared the percentage of medical travelers that fall into poverty annually between a sample of subsidized travelers and non-subsidized travelers. We focused on measuring equality of treatment of all with respect to public subsidy for medical travel instead of equity which promotes fairness. Findings from this study will guide countries facing similar problems of limited capacity or lack of economies of scale in ways to alleviate poverty due to health care payments.

## Methods

### Study design

The data are derived from a cross-sectional survey of inbound Maldivian patients who travelled for medical treatment during the period June–December 2013.

### Study setting

**Fig. 1:**
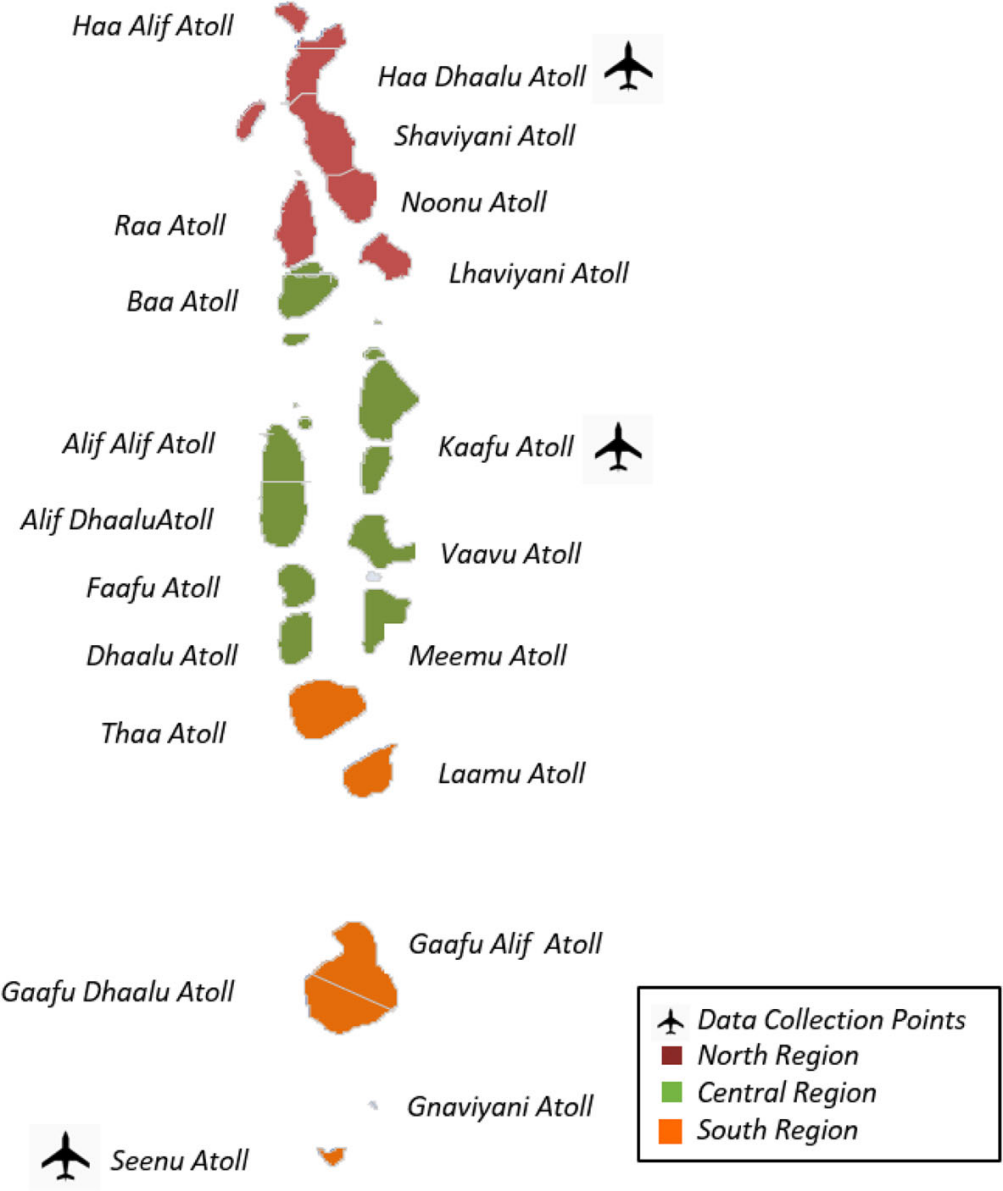
Data collection points on the map of Maldives

The study was undertaken in the Republic of Maldives, situated in the Indian Ocean, and neighboring Sri Lanka and India – the two countries that the majority of Maldivian medical travelers frequent [8]. At the time of the study, three international airports (one in the North, Center and South) were the exit points for travel abroad. Figure 1 shows the data collection points on the map of the Maldives.

### Sample size

A minimum sample-size of 342 subsidized and 473 non-subsidised travellers was calculated as required to estimate expenditures with a precision of at most 5% difference from the average actual expenditure in each group and with the assumption of a 30% non-response rate. During the study period, we were able to interview 344 subsidised and 471 non subsidised travellers who were included in the analysis.

### Sampling

We used multiple sources to reach medical travellers. Subsidised travellers were recruited from the database of medical travellers maintained by the government of the Maldives. Among 2556 subsidized patients who travelled during the study period, 344 were randomly selected and a telephone interview was conducted. Non-subsidized travellers were recruited by surveying the three international airports of Maldives (Hanimaadhoo international airport in the North, Ibrahim Nasir international airport in the central region and Gan international Airport in the south) and the regional health facility in each of the three regions. Face-to-face interviews were conducted with a consecutive sample of 335 travellers at the airports while telephone interviews were conducted with 136 available travellers contacted from the lists of referrals abroad at the regional health facilities. A total of 473 self-funded patients were interviewed by the end of the study period.

### Study sample

There were subsidized and non-subsidized cases. A subsidized patient was defined as one if any part of his/her expenses for medical travel was provided by the government. Proxies were allowed for the elderly, young or very ill patients. Traditional medicine centers and facilities not registered as a health facility in the destination country were excluded.

### Data collection

Data were collected from August to December 2013. All 815 participants recruited from all the sources were interviewed using a common questionnaire which was pre-tested and researcher-administered. The questionnaire was translated into the local language (Dhivehi) and official approval for the translation was sought from the Ministry of Education of Maldives. Data on demographic and household characteristics, monthly OOP household expenditures, disease profile and costs of the last medical travel episode were collected.

#### Data management

All monetary values were expressed in purchasing power parity (PPP) dollars at the conversion rate of 11.1 Mrf per PPP dollar for the year 2013. [9] The PPP conversion factor is the number of units of a country’s currency required to buy the same amount of goods and services in the domestic market as a U.S. dollar would buy in the United States [10]. The PPP exchange rate was used throughout the analysis as it helps to minimize misleading international comparisons that normally arise with the use of market exchange rates. Monthly household income was used as a living standard measure in the study. Monthly household income was grouped into quintiles among the subjects.

The main components of the consumption aggregate include monthly OOP expenses for health, food and non-food expenses multiplied by 12 to derive annual consumption aggregates. Health payments comprise of sub-aggregates for both local expenses and medical expenses born during the last episode of medical treatment abroad multiplied by the number of visits each year. Likewise food and non-food expenses born during the medical travel episode were represented in the respective sub aggregates. Food consumption includes purchases made from income sources, while non-food consumption includes items such as clothing, education, travel, rent energy. Following standard practice, lumpy expenditures such as consumer durable goods, constructions, fines and debt were excluded.

The primary diagnosis for which treatment was sought abroad was coded under the 22 major groupings of ICD 10, version 2010. Ailments or descriptions that did not fall under any major disease group were coded under “Factors influencing health status and contact with health services” (Z00-Z99 of ICD10). One hundred and sixty nine observations in this group consisting of 65 subsidized and 104 non-subsidized travelers were omitted in the analysis of the disease profile, following WHO advice not to use this group for comparisons. [11] To measure disease specific impoverishment, diseases were sorted by the median medical cost of treatment and the top five diseases were analyzed.

Residential atolls of the travelers were first grouped into the 7 administrative provinces and were regrouped by collapsing the Upper North and North provinces into North region, North central and central province into Central region and South central, Upper south and South provinces in to South region. Regrouping was done to manage the small number of records at province level that did not allow for proper analysis.

### Statistical analysis

The household was the unit of analysis in this study. Data analysis was conducted using the open source R software, version 3.1.0 [12]. We measured both inequality and poverty. Inequality measurements include basic dispersion measures such as the Pen’s Parade, percentile ratio, consumption share of the poorest using frequency distribution across quintiles. Non-parametric tests were used to identify differences in the patterns and the level of significance was set at *p* < 0.05. As annual income was ranked into quintiles and did not follow normality, the Kruskal Wallis test, chi-square test and the Ranksum test were used to allow analysis against other nominal values. For the analysis of impoverishment, we used the international poverty lines of $2. Poverty head count^1^ and poverty gap^2^ were measured following O’Donnell et al. [13].

If ***x***_***i***_ is the household *i*’s consumption per capita, ${\boldsymbol{p}}_{\boldsymbol{i}}^{\boldsymbol{gross}}$ =1, if ***x***_***i***_ < the poverty line, and ${\boldsymbol{p}}_{\boldsymbol{i}}^{\boldsymbol{gross}}$ =0, if otherwise.

The Gross poverty head count and gross poverty gap is expressed as

$$ {\boldsymbol{H}}^{\boldsymbol{gross}}={\sum}_{\boldsymbol{i}=1}^{\boldsymbol{N}}{\boldsymbol{s}}_{\boldsymbol{i}}{\boldsymbol{p}}_{\boldsymbol{i}}^{\boldsymbol{gross}}/{\sum}_{\boldsymbol{i}=1}^{\boldsymbol{N}}{\boldsymbol{s}}_{\boldsymbol{i}} $$

$$ {\boldsymbol{G}}^{\boldsymbol{g}\boldsymbol{ross}}={\sum}_{\boldsymbol{i}=1}^{\boldsymbol{N}}{\boldsymbol{s}}_{\boldsymbol{i}}{\boldsymbol{g}}_{\boldsymbol{i}}^{\boldsymbol{g}\boldsymbol{ross}}/{\sum}_{\boldsymbol{i}=1}^{\boldsymbol{N}}{\boldsymbol{s}}_{\boldsymbol{i}} $$

Where *s*_*i*_ is the size of the household, *N* is the number of households in the sample and *g*^*gross*^ = ${p}_i^{gross}$ (poverty line- *x*_*i*_). The net of health payments poverty gap *g*^*net*^ = ${p}_i^{net}$ (poverty line–(*x*_*i*_ − *T*_*i*_)), where *T*_*i*_ is the per capita out of pocket spending on health.

The impact of poverty was derived by assessing the difference between gross and net of OOP payments to health care in each of the above measures.

$$ \mathrm{Poverty}\ \mathrm{head}\ \mathrm{count}={\boldsymbol{H}}^{\boldsymbol{gross}}-{\boldsymbol{H}}^{\boldsymbol{net}} $$

$$ \mathrm{Poverty}\ \mathrm{gap}={\boldsymbol{G}}^{\boldsymbol{gross}}-{\boldsymbol{G}}^{\boldsymbol{net}} $$

Jan Pen’s parade [13] .for household expenditures gross and net of OOP payments to health care was used to plot health adjusted impoverishment among households of medical travelers. The international poverty line of $2 per day was used to define poverty on the parades. The effect of health payments on the Pen’s Parade of per capita household expenditure was illustrated using the ‘Paint drip’ chart. Each “paint drip,” represents a household showing the extent to which the subtraction of health payments reduces consumption. A bar that crosses the poverty line implies that a household is not counted as poor on the basis of gross consumption but is poor on the basis of net consumption [13]

## Results

Table 1 shows the utilization of medical travel across income quintiles. Medical travelers were mostly from the middle-income group (Quintile 3) with no statistical difference between subsidized and non-subsidized travelers (*p* value: 0.499). Among regions, the number of medical travelers was lowest among the poorest people in the Central region, while in both North and South regions, the share of the poorer quintile was highest. Most of the travelers had ownership of their homes, while of people living in rented housing the poorer people were less likely to travel abroad (*p* value: 0.001). Among the poorest quintile, travel was most common among smaller families.

**Table 1:** Utilization of medical travel across income quintiles

Indicator	Q1	Q2	Q3	Q4	Q5	overall	*p* value	Ratio of Q1:Q5
All travelers	172	154	227	137	125	815		
Financial protection							0.499	
Subsidized travelers (*N* = 344)	81 (23.5)	65 (18.9)	93 (27)	59 (17.2)	46 (13.4)	344 (100)		1.8
Non subsidized travelers (*N* = 471)	91 (19.3)	89 (18.9)	134 (28.5)	78 (16.6)	79 (16.8)	471 (100)		1.1
Region of residence							< 0.001	
Central region *N* = 366	40 (10.9)	57 (15.6)	106 (29)	80 (21.9)	83 (22.7)	366 (100)		0.5
North region *N* = 245	69 (28.2)	54 (22)	71 (29)	27 (11)	24 (9.8)	245 (100)		2.9
South region *N* = 204	63 (30.9)	43 (21.1)	50 (24.5)	30 (14.7)	18 (8.8)	204 (100)		3.5
Type of tenure							< 0.001	
Rent free *N* = 63	17 (27)	13 (20.6)	20 (31.7)	9 (14.3)	4 (6.3)	63 (100)		4.3
Rented *N* = 147	7 (4.8)	23 (15.6)	55 (37.4)	41 (27.9)	21 (14.3)	147 (100)		0.3
Owner occupied *N* = 605	148 (24.5)	118 (19.5)	152 (25.1)	87 (14.4)	100 (16.5)	605 (100)		1.5
Household size							< 0.001	
Small (≤5 members)	131 (27.4)	91 (19)	146 (30.5)	65 (13.6)	45 (9.4)	478 (100)		2.9
Medium (6–10 members)	38 (15.3)	46 (18.5)	68 (27.3)	52 (20.9)	45 (18.1)	249 (100)		0.8
Large (> 10 members)	3 (3.4)	17 (19.3)	13 (14.8)	20 (22.7)	35 (39.8)	88 (100)		0.1
Length of stay (Median, IQR)	12.5 (9.8,19)	15 (10,20)	15 (10,20)	14 (10,21)	12 (10,21)	14 (10,20)	0.356	1.0
Number of visits (Median, IQR)	1 (1,1.2)	1 (1,2)	1 (1,1)	1 (1,2)	1 (1,1)	1 (1,1)	0.374	1.0
Age (Median, IQR)	48 (36,62)	40.5 (21.2,50)	40 (27.5,53)	37 (20,50)	42 (22,55)	42 (25,54)	< 0.001	1.6

Note: *P* values are from Kuskal-Wallis and chi squared tests

Table 2 shows that medical expenditure abroad was similar across all income quintiles (p value: 0.95). There was no statistical difference in OOP expenditures on medical purposes abroad and annual OOP expenditure on health was similar across income quintiles. The government subsidy across income quintiles was also similar indicating that the health financing structure is not pro poor. Expenditure on food and non-food items was significantly different between the income quintiles, with non-medical expenditure lowest in the poorest quintile (p value: 0.025).

**Table 2:** Expenditure inequalities among medical travelers

Indicator	Income quintiles						p value (between quintiles)
	Q1	Q2	Q3	Q4	Q5	Overall	
Total (N)	172	154	227	137	125	815	
Medical expenditure abroad (USD PPP)	670.4 (268.2,1566.8)	670.4 (257.4,1340.9)	603.4 (223.9,1633.8)	626.2 (205.2,1475)	536.3 (211.9,1340.9)	670.4 (243.4,1542)	0.952
Non-medical expenditure abroad (USD PPP)	2274.8 (1644.2,3111.8)	2516.8 (1794.8,4062.8)	2695.1 (1749.2,4026.6)	2449.8 (1652,3531.9)	2650.9 (1717.7,3763.8)	2481.9 (1711.6,3731.6)	0.025
OOP medical expenditure abroad (USD PPP)	321.1 (64,927.5)	293 (59,804.5)	272.2 (67,804.5)	348.6 (101.9914.5)	284.3 (73.7804.5)	295 (67,871.6)	0.984
Medical expenditure by government abroad (USD PPP)	0 (0,268.2)	0 (0,477.7)	0 (0,592.7)	0 (0,434.4)	0 (0,234.7)	0 (0,418.4)	0.963
Annual OOP household expenditure on health (USD PPP)	1158.5 (473,2536.6)	1151.8 (512.9,3261.3)	1224.2 (431.8,2781)	1469.6 (690.5,3137.6)	1627.8 (429.1,3727.6)	1311.4 (513.6,3118.9)	0.369
Annual OOP household expenditure on food (USD PPP)	3372.3 (2364,4890.2)	5127.5 (3248.9,6527.4)	5261.6 (3719.6,8165.9)	6259.2 (3856.3,8595)	8418 (5571.3,11,126.5)	5213.3 (3381.7,7571.9)	< 0.001
Annual OOP household expenditure on non-food (USD PPP)	2543 (1527.3,4349.1)	3811.4 (2448.4,7327.2)	4081.6 (2677.7,8495.1)	5492.2 (2551.7,11,786.2)	4355.1 (2015.3,8887.3)	3810.8 (2174.2,7964.8)	< 0.001

Note: P values are from Kuskal-Wallis and Ranksum tests

As Table 3 shows, the median cost of treatment was highest among the patients who sought treatment for neoplasms. The percentile ratio shows that for every dollar spent by the poorest quintile on the treatment of neoplasms, the richest quintile spent 114 dollars. The impoverishment effect from neoplasms was found to be very low compared to other diseases which may be explained by the high proportion of people subsidized for this treatment (86.3%), but the impoverishment effect was lowest among the patients treated for diseases of the nervous system (2%), for which only one third obtained the public subsidy. Across all of the top five diseases, the expenditure share of the poorest percentile was markedly lower. Diseases of the circulatory system, eye and musculoskeletal system had the most impoverishing effect where only half or fewer of the patients had received the subsidy.

**Table 3:** Dispersion ratio across the top five diseases among medical travelers, 2013

Disease Group (ICD 10)		Medical cost during last episode of treatment abroad	Richest 5%	Poorest 5%	Percentile ratio	Poverty head count ratio			Proportion of financial protection
	N	Median (IQR)	95th percentile	5th percentile		Gross	Net	Difference	%
Diseases of the circulatory system	82	926.54 (266, 2625)	12,128.9	124.7	97.3	3%	18%	16%	57.3
Diseases of nervous system	69	795.13 (305, 1474)	7237.2	127.9	56.6	0%	2%	2%	33.3
Diseases of musculoskeletal system	65	603.39 (218, 1072)	2111.9	73.2	28.8	6%	16%	10%	15.4
Diseases of genitourinary system	62	808.54 (305, 1331)	4009.2	67.1	59.7	10%	18%	8%	54.8
Diseases of the eye & adnexa	51	492.09 (156, 1149)	2136.0	52.3	40.8	17%	28%	11%	54.9
Neoplasms	51	1193.37 (412, 4312)	9589.2	83.8	114.4	2%	7%	5%	86.3

**Fig. 2:**
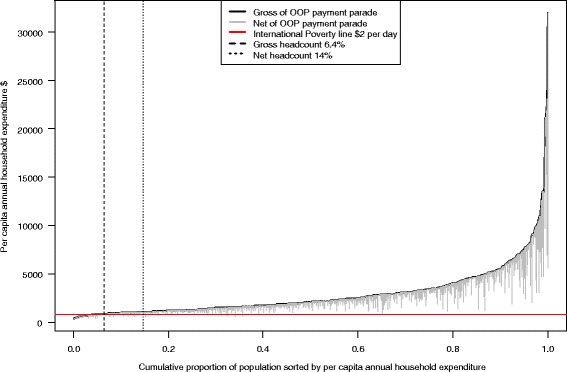
Effect of Health payments on Pen’s Parade of annual household expenditures among medical travelers

Figure 2 shows the Pen’s Parade for household expenditure before making OOP expenditures to healthcare, with the paint drips representing the effect on expenditure for each household after making OOP payments to health. Annually 6 and 14% of medical travellers in the Maldives fall into poverty (an income of less than $2 per day) after making OOP payments to health care. Distribution of health payments were found to be proportionate to total consumption, with larger health payments at higher values of total consumption and smaller among the households in the lower half of the distribution. Although expenditure on health is lower among the households in the lower half of the distribution, they are the households most likely to be pushed below the poverty line by health payments. The household expenditure net of health payments between subsidised and non-subsidised patients indicated that 17% of the subsidized travellers and 12% of the non-subsidised patients fall below poverty line after making payments to health care.

## Discussion

Contrary to conclusions in much of the existing literature on medical travel, most of the socio demographic indicators, and costs of treatment abroad among these Maldivian medical travelers, were similar across different household income levels [14–16]. Medical expenditure abroad and OOP expenditures on health were consistent among the poor and the rich irrespective of government subsidy. This suggests that the Maldivian health financing structure supports equality rather than being equity-sensitive. Study results pointed towards disease specific inequalities and impoverishment among medical travelers, where the expenditure share of the poorest percentile was pointedly lower across all the top five diseases. Although patients suffering from neoplasms bear the highest cost of treatment abroad, the level of impoverishment was low. Disease groups which had the highest levels of impoverishment had only half of the patients covered by the public subsidy. Yet, the disease group with the lowest impoverishment level had only one third of its patients covered by the public subsidy. This indication of an association between the government subsidy and impoverishment needs to be further explored. Overall, the type of disease for which the patient travelled seemed a greater predictor of impoverishment than the government subsidy. Study findings also showed that more of the subsidized patients fell into poverty compared to the non-subsidized which offers important directions for the revision of the current benefit package and to implement appropriate targeting mechanisms. Taken together, these findings suggest that the current government subsidy has little effect and does not offer protection from impoverishment.

Utilization of medical travel was more common among middle-income earners and the poorer quintiles across both subsidized and non-subsidized travelers. The predominant public financing for health care by the government of Maldives and availability of other sources of insurance with specific targeting mechanisms for medical travel may explain the pro poor utilization pattern. By 2013, the government of the Maldives spent 744,921,545 MVR (approximately USD 48,308,790) under the universal health care program ‘Aasandha’, and an additional 40,484,849MVR (approximately USD 2,625,476) under the emergency medical welfare program. Other large public institutions such as the police service and the defense force offer health insurance for their employees and their families [17]. Due to the predominance of public funding towards health coverage, only three parties in the private sector targets the remaining population with offers of medical travel included [18].

The study findings contradict much of the existing literature on medical travel which has highlighted the possibility for inequalities in access created by medical travel [19–23]. However, our findings are congruent with the achievements of UHC experienced in Thailand [24], Mexico [25] and Turkey [26] which has improved access to the poor within the domestic health system. This study provides support that a public funded UHC can promote access to health services abroad for the poor as well.

Medical expenditures abroad were similar across all income quintiles of medical travelers and with no statistical difference in OOP expenditures on medical purposes abroad and annual OOP expenditure on health across income quintiles. Distribution of the government subsidy across income quintiles was also similar indicating that the health financing structure is not equity-sensitive but focused on maintaining equality. The 2008 report of the WHO Commission on Social Determinants of Health states that inequities in health results from the unequal distribution of power, prestige and resources [27]. According to the World Bank country snapshot of March 2014, the Maldives endures a high level of inequality at a national Gini-coefficient of 0.37 which explains the government policy [28]. At the same time the same country report highlights that almost a quarter of the population (24%) lives below the international poverty line of $2 per day and the study results are in line with this as 14% of the travelers in the study were impoverished after making payments to health care. Study findings are suggestive that a balance of equality and equity is important in order to improve access to the whole population and maintain fairness.

We observed disease-specific inequalities and impoverishment among medical travelers. Across all the top five diseases, the expenditure share of the poorest percentile was pointedly lower. The median cost of treatment was highest among the patients who sought treatment for neoplasms and the percentile ratio showed that for every dollar spent by the poorest quintile on the treatment of neoplasms, the rich spent 114 dollars. A multivariate analysis that assessed the socioeconomic-differentials in the impact of OOP expenditure on impoverishment in China and India indicated that lower standard of living increases the odds of falling below poverty line significantly [29]. The findings direct policy interventions to prioritize patients with lengthy and costly treatments, based on socioeconomic indicators of the patients. Diseases of the circulatory system, eye and musculoskeletal system had the most impoverishing effect where only half or fewer of the patients had received the subsidy. Future research questions need to explore the reasons why people do not access government subsidy and the reasons for the existence of disease-specific impoverishment.

Impoverishment was found to be higher among the subsidised travellers with 17% of the subsidized travellers falling below poverty line after making payments to health care compared to 12% of the non-subsidised patients. The current level of government investment and current offer of subsidy package did not prove to be sufficient to protect some beneficiaries from impoverishment. Consistent with the study findings, a systematic review of studies reporting on the impact of health insurance schemes that were intended to benefit the poor showed that there was some evidence that health insurance may prevent high levels of expenditure but it did not show any correlation with high enrolment in the schemes with better outcomes [30]. Catastrophic spending in low- and middle-income countries has been shown to be positively and strongly correlated with the availability of health services in the country and with ageing populations [31]. This is applicable to the study setting where the main reason for medical travel has been due to unavailability of health services in the country. Government subsidy can be an important mechanism to protect households from impoverishment when appropriate targeting mechanisms are applied.

### Strengths and limitations

The sample was derived using a cross-sectional survey via multiple sources, which helped to acquire many medical travellers in a short period of time. However it may have contributed to sampling bias. To avoid estimation bias, stratified samples of subsidized and non-subsidized travellers for the strata sizes were calculated which also allowed the application of different measurement techniques such as telephone interviews and face to face interviews whichever was most viable for each stratum. The quality of the consumption data on households is weak as the sub aggregates were too limited. Data on detailed consumption sub aggregates would have provided more information such as expenditures on inpatient care, outpatient, and drugs instead of a total health expenditure value. In addition, data were based on self-reports which could not be verified and which may have led to under reporting of income and over reporting of expenditures.

## Conclusion

Evidence of a strong association between predominant public financing of medical travel and equality was found. With universal eligibility to government subsidy for medical travel, utilization of treatment abroad, medical expenditures abroad and OOP expenditures on health among Maldivian medical travellers were similar between the poor and the rich. We conclude mixed evidence on the linkages between public financing of medical travel and impoverishment. Despite the fact that they bear the highest cost of treatment abroad, very low levels of impoverishment were observed among patients seeking treatment for neoplasms, for which more than 85 % of the patients received the government subsidy. In comparison, other disease groups had high levels of impoverishment where the number of beneficiaries was comparatively low. However, the proportion of subsidised travellers suffering from impoverishment was higher than that of the non-subsidized group which calls for more evidence to identify reasons why people do not seek the public subsidy for medical travel and the health seeking behaviours of the subsidized and the non-subsidized traveller. There is need for further research to examine differences in impoverishment levels between households with and without medical travel and investigate the relationship between public subsidy for medical travel and impoverishment.

## Footnotes

1 The poverty headcount measures the proportion of the population that is poor, defined as falling below the poverty line.

2 The poverty gap measures the extent to which individuals fall below the poverty line, measuring the depth of poverty as well as the incidence line [13].

## References

[1] WHO. What is universal health coverage? 2016 [15 May 2016]; Online Questiona and answer archives]. Available from: http://www.who.int/features/qa/universal_health_coverage/en/.

[2] Wong KM, Velasamy P, Arshad TNT, editors. Medical tourism destination SWOT analysis: A case study of Malaysia, Thailand, Singapore and India. SHS Web of Conferences; 2014: EDP Sciences.

[3] Peng Y, Chang W, Zhou H, Hu H, Liang W (2010). Factors associated with health-seeking behavior among migrant workers in Beijing, China. BMC Health Serv Res.

[4] Zafar SY, Peppercorn JM, Schrag D, Taylor DH, Goetzinger AM, Zhong X (2013). The financial toxicity of cancer treatment: a pilot study assessing out-of-pocket expenses and the insured cancer patient's experience. Oncologist.

[5] Mataria A, Raad F, Abu-Zaineh M, Donaldson C (2010). Catastrophic healthcare payments and impoverishment in the occupied Palestinian territory. Appl. Health Econ. Health Policy.

[6] Wagstaff A, van Doorslaer E (2003). Catastrophe and impoverishment in paying for health care: with applications to Vietnam 1993–1998. Health Econ.

[7] Limwattananon S, Tangcharoensathien V, Prakongsai P (2007). Catastrophic and poverty impacts of health payments: results from national household surveys in Thailand. Bull World Health Organ.

[8] Authority MM. Maldivians travelling Abroad 2013. Republic of Maldives: Maldives Central Bank; 2014.

[9] National Bureau of statistics M. Statistical Year Book of Maldives 2015. Republic of Maldives 2015.

[10] Group TWB. Data: PPP conversion factor, private consumption (LCU per international $). 2015 [cited 2015 08 December]; World Bank, International Comparison Program database.]. Available from: http://data.worldbank.org/indicator/PA.NUS.PRVT.PP.

[11] WHO. (ICD-10) Version for 2010. 2014 [30 July 2014]; Available from: http://apps.who.int/classifications/icd10/browse/2010/en#/XXI

[12] Team RC (2012). R: a language and environment for statistical computing.

[13] O'Donnell OA, Wagstaff A. Analyzing health equity using household survey data: a guide to techniques and their implementation. Washington DC: World Bank Publications; 2008.

[14] Lunt N, Horsfall D, Hanefeld J. Handbook on Medical Tourism and Patient Mobility. UK: Edward Elgar Publishing; 2015.

[15] Gan LL, Frederick JR (2013). Medical tourists: who goes and what motivates them?. Health Mark Q.

[16] Yu JY, Ko TG (2012). A cross-cultural study of perceptions of medical tourism among Chinese, Japanese and Korean tourists in Korea. Tour Manag.

[17] Health Mo. Maldives National Health Accounts 2009–2011. Republic of Maldives: National Social Protection Agency Maldives (NSPA); 2013.

[18] International Business Publications U. Maldives investment and business guide: strategic and practical information. [Place of publication not identified]: Intl Business Pubns Usa; 2015.

[19] Connell J (2011). A new inequality? Privatisation, urban bias, migration and medical tourism. Asia Pac Viewp.

[20] Johnston R, Crooks VA, Snyder J, Kingsbury P (2010). What is known about the effects of medical tourism in destination and departure countries? A scoping review. Int J Equity Health.

[21] Gupta AS (2008). Medical tourism in India: winners and losers. Indian J Med Ethics.

[22] Smith R, Álvarez MM, Chanda R (2011). Medical tourism: a review of the literature and analysis of a role for bi-lateral trade. Health Policy.

[23] Whittaker A, Manderson L, Cartwright E (2010). Patients without borders: understanding medical travel. Med Anthropol.

[24] Limwattananon S, Prakongsai P, Tangcharoensathien V. The equity impact of Universal Coverage: health care finance, catastrophic health expenditure, utilization and government subsidies in Thailand. Consortium for Research on Equity in Health Systems (CREHS) Report. 2011.

[25] Knaul FM, González-Pier E, Gómez-Dantés O, García-Junco D, Arreola-Ornelas H, Barraza-Lloréns M (2012). The quest for universal health coverage: achieving social protection for all in Mexico. Lancet.

[26] Atun R, Aydın S, Chakraborty S, Sümer S, Aran M, Gürol I (2013). Universal health coverage in Turkey: enhancement of equity. Lancet.

[27] Health CoSDo. Closing the gap in a generation: health equity through action on the social determinants of health: final report of the commission on social determinants of health. 2008. 10.1016/S0140-6736(08)61690-6 18994664

[28] Bank TW. Maldives: Country Snapshot October 2014. 2014.

[29] Kumar K, Singh A, Kumar S, Ram F, Singh A, Ram U (2015). Socio-economic differentials in impoverishment effects of out-of-pocket health expenditure in China and India: evidence from WHO SAGE. PLoS One.

[30] Acharya A, Vellakkal S, Taylor F, Masset E, Satija A, Burke M, Ebrahim S. The Impact of health insurance schemes for the informal sector in low- and middle-income countries: a systematic review. Policy research working paper; No. 6324. Washington DC: World Bank; 2013. https://openknowledge.worldbank.org/handle/10986/12163.

[31] Xu K, Evans DB, Carrin G, Aguilar-Rivera AM, Musgrove P, Evans T (2007). Protecting households from catastrophic health spending. Health Aff.

